# Nutrients in Infancy: Progress and Prospects

**DOI:** 10.3390/nu9101131

**Published:** 2017-10-17

**Authors:** Colin Binns, Mi Kyung Lee, Masaharu Kagawa

**Affiliations:** 1 Faculty of Health Sciences, School of Public Health, Curtin University, Perth 6845, Australia; 2 School of Health Professions, Murdoch University, Perth 6150, Australia; m.k.lee@murdoch.edu.au; 3 Institute of Nutrition Sciences, Kagawa Nutrition University, Saitama 350-0288, Japan; mskagawa@eiyo.ac.jp

This monograph, based on a special issue of Nutrients, contains 31 papers—5 reviews and 26 original publications—that reflect the wide spectrum of current research on nutrients and infancy. The papers include populations from many countries, including Australia, Canada, China, Ireland, Italy, Malaysia, Mexico, Netherlands, Norway, Singapore, Spain, Sweden, Thailand, the United Kingdom and the USA. The largest group of papers are on the nutrient composition of breastmilk and the timing and factors that influence the nutrient content of breastfeeding. The age range of the subjects studied was from preterm very low birth weight babies to older infants.

There are two key objectives for infant nutrition: survival, and to lay foundations for growth and development that will optimise health throughout a long lifespan. Besides the ethical problems associated with infant research, there is the obvious difficulty of conducting lifespan research in humans [1]. Continued infant nutrition research will rely on retrospective epidemiological studies and the increasing knowledge of health and lifespan biomarkers.

In compiling this special issue, there were two issues that the editors found to be important. Definitions have always been central to successful research, and the definition used determines the results presented in relation to breastfeeding and complementary feeds. Much has been written about definitions of breastfeeding, but there is still little standardisation in publications [2,3,4]. At the very least, authors should adhere to the standard WHO (World Health Organisation) definitions of exclusive breastfeeding, but then describe what they have actually done to allow others to accurately interpret their results.

The ethics of research are always important in all publications, but are particularly so in the area of infant nutrition. The editors of this monograph summarised the ethical principles involved in infant nutrition research [5]. In recent years, the influence of early nutrition on later health and longevity has been increasingly studied. This means that extra care must be taken with any early life intervention studies.

One third of the articles are on breastmilk composition from countries around the globe, and several more are on factors associated with breastfeeding duration. This reflects the centrality of breastmilk to the supply of nutrients in infancy, its importance for lifelong health and development, and the fact that we still have a great deal to learn. The results of several studies have implications for public health nutrition programs. It is estimated that 1.9 billion people live in areas of the world subject to subclinical iodine deficiency, which is important for cognitive development in infancy [6]. The study by Jorgenson and her colleagues demonstrates that, in Australia, with a food fortification program and the recommendation that pregnant women take 150 mcg iodine supplements daily, the iodine content of breastmilk is generally adequate [7].

In the NHANES (National Health and Nutrition Examination Survey) study from the USA, an analysis of NHANES data on iron, calcium, and zinc among children in the second year of life using two days of dietary intake data found that one in four children and one in ten children had usual intakes below the RDA (Recommended Dietary Allowance) for iron and calcium, respectively. [8].

The relationship between nutrients and growth will be a continued area of interest. The prevalence of stunting is still relatively high in some world regions and is a priority of the UN Sustainable Development Goals (UNSDGs) [9]. At the other end of the continuum of development, child obesity is now a major problem, and is also an important target [9]. Micro nutrient deficiency may influence stunting and undernutrition, but more research is still required to complete our understanding. The review on iron and zinc supplementation found that low doses of daily iron and zinc use between 6 and 23 months of age had a positive effect on child’s iron and zinc status [10]. However, this did not translate into a reduction in the proportion of children with stunting. Further research is required into stunting to achieve the UNSDGs. The role of protein in over-nutrition and the development of obesity has been a fertile area of research [11]. The lower protein level of breastmilk compared to the higher protein levels of many infant formulae provides a biological basis for understanding the protective effects of breastmilk against obesity [12].

An area of continued interest is the relationship between the human microbiomes and nutrition. In 2007, the National Institutes of Health launched the Human Microbiome Project to promote research into the ways in which health outcomes are linked to changes in the microbiome [13,14]. The human microbiome is exceedingly complex, both in its composition and in its interrelation with nutrients. Nutrients can change the nature of the microbiome and in turn the microbiome has effects on metabolism [15]. It is now well understood that infant feeding method, mechanism of delivery and the use of perinatal antibiotics can influence the composition of the microbiome [16]. One of the mechanisms responsible for protection against obesity provided by breastfeeding may be through the development of a healthy microbiome [17]. There is increasing evidence of links between early development (particularly in the perinatal period) and later mental illness [18,19]. Again this linkage may be through changes to the human microbiome [20,21,22]. The use of pre-lacteal feeds and complementary feeds (i.e., not exclusively breastfeeding), C-section delivery and use of antibiotics (particularly in the perinatal period) changes the composition of the microbiome [23,24]. From a public health perspective, the increasing rates of operative delivery, widespread antibiotic use and low rates of exclusive breastfeeding may be associated with increased rates of obesity, diabetes and other metabolic disorders and mental illness. Exclusive breastfeeding remains as important as ever as the basis of infant and maternal health [25]. As long-term changes to the microbiome may be one mechanism by which early life dietary intake can modify health in later life, research into nutrients and microbiome health will be important.

We believe this collection is a useful summary of progress in many areas of infant nutrition. It also points to many research needs, in order to better understand infant nutrient requirements, growth and healthy development. With the present rate of progress, it may only be a few years before another volume is required.
